# The macrophage-associated microRNA-4715-3p / Gasdermin D axis potentially indicates fibrosis progression in nonalcoholic fatty liver disease: evidence from transcriptome and biological data

**DOI:** 10.1080/21655979.2022.2072602

**Published:** 2022-05-06

**Authors:** Shuai Chen, Xiurong Cai, Yu Liu, Yu Shen, Adrien Guillot, Frank Tacke, Liming Tang, Hanyang Liu

**Affiliations:** a Center of Gastrointestinal Disease, The Affiliated Changzhou No. 2 People’s Hospital of Nanjing Medical University, Changzhou, Jiangsu, P.R. China; b Department of Hematology, Oncology and Tumor Immunology (CVK), Charité Universitätsmedizin Berlin, Berlin, Germany; c Institute of Radiology (CVK), Charité Universitätsmedizin Berlin, Berlin, Germany; d Cell Biology, Deutsches Rheuma-Forschungszentrum Berlin (DRFZ), a Leibniz Institute, Berlin, Germany; e Department of Hepatology and Gastroenterology (CVK), Charité Universitätsmedizin Berlin, Berlin, Germany

**Keywords:** NAFLD progression, fibrosis, macrophage, miR-4715-3p/*GSDMD* axis, cell death

## Abstract

Nonalcoholic fatty liver disease (NAFLD) is highly possible to progress to cirrhosis, malignancy, and liver failure through fibrogenesis. The enormous potential of pathogenetic and therapeutic targets in NAFLD has been revealed. This study aimed to explore novel factors potentially indicating or mediating NAFLD progression. Multiple bulk and single-cell RNA sequencing datasets were used, in which landscapes of cell populations were clarified to characterize immune cell infiltration. Significantly high infiltration of macrophages (MPs) was discovered during NAFLD progression. Samples in bulk NASH datasets were regrouped by MP level. Highly differentially expressed genes (DEGs) were identified in the Ctrl vs. NASH comparison, low MP vs. high MP comparison, and the weighted gene co-expression network analysis (WGCNA) clusters. Eight hub genes were identified as promising targets by protein–protein interaction analysis and validated in fibrosis progression, microRNA (miR)–protein interactions were predicted, and the hub genes were verified in a free fatty acid (FFA)-induced macrophage injury model. The results showed that Gasdermin D (*GSDMD*) was upregulated with fibrosis progression in NAFLD and was associated with macrophage infiltration. In addition, a potential regulator (miR-4715-3p) was correlated with *GSDMD*. The miR-4715-3p/*GSDMD* axis potentially modulates macrophage-associated immunity and indicates fibrosis progression in NAFLD.

## Graphical Abstract

**Figure:**
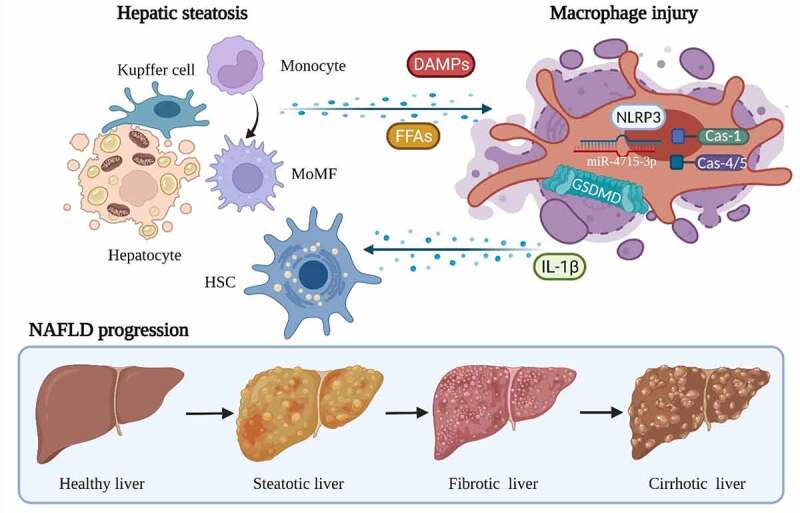

## Highlight

1. Macrophage infiltration is potentially correlated with NAFLD progression.

2. Macrophage-associated key gene GSDMD is upregulated in NAFLD progression.

3. has-miR-4715-3p and GSDMD are parallelly activated in fatty acid-induced macrophage injury.

4.has-miR-4715-3p/GSDMD axis potentially modulates macrophage-associated immunity and indicates fibrosis progression in NAFLD.

## 1. Introduction

onalcoholic fatty liver disease (NAFLD) is one of the most common chronic liver diseases worldwide. Lipid deposition and steatosis are independent causes of NAFLD, independent of drug-related, alcohol-related, or genetic risks. The pathogenesis and progression of NAFLD are fundamentally mediated by the complex interactions among steatosis, fibrosis, and inflammation [1]. Although early symptoms rarely appear in nonalcoholic steatohepatitis (NASH) patients, chronic liver cirrhosis eventually leads to liver failure or hepatocellular carcinoma (HCC). Notably, multiple types of immune modulation occur during NAFLD progression. As executors and directors in the immune microenvironment, immune cells, especially macrophages and T cells, are requisite factors in the pathogenesis, cirrhogenesis, metabolic compensation, and progression of NAFLD [2,3].

In almost all chronic liver diseases, fibrosis occurs and progresses with inflammatory reactions, which are involved in liver macrophage modulation. Systematically, the liver macrophage population generally consists of Kupffer cells (KCs) and monocyte-derived macrophages (MoMFs). Liver macrophages play an essential role in fibrogenesis through interactions with hepatic stellate cells (HSCs) [4]. As a feedback regulatory mechanism, chemokines and cytokines secreted by HSCs enhance macrophage infiltration and expansion, which promote the fibrotic phenotypes and survival of HSCs. Passively activated M2 macrophages are associated with liver injury in NAFLD and mediate fibrotic responses conducive to liver remodeling and regeneration by secreting transforming growth factor-β (TGF-β) and platelet-derived growth factor (PDGF) [5]. Although crucial roles of macrophages in NAFLD and other inflammatory liver diseases have gradually been revealed, the exact mechanisms of macrophage involvement in the pathogenesis and progression of these diseases remain unclear. In recent decades, transcriptome analysis was rapidly increased and has provided logical and scientific guidance to traditional biological studies. Fundamentally, computational analysis based on high-throughput datasets can indicate both target genes and biological processes regarding specific research goals [6,7]. Therefore, in NAFLD with a chronic fibrosis progression, bioinformatics analysis would bring out unique advantages to understand disease characteristics and therapeutic targets.

The hypothesis of this study is that miR-4715-3p/*GSDMD* axis associating macrophage infiltration potentially indicates NAFLD progression. Based on the analysis of high-throughput bulk/single-cell RNA sequencing datasets, this study aims to reveal immune cell landscapes in NAFLD livers during fibrosis progression. Thereby, through further MP-associated analyses, the miR-4715-3p/*GSDMD* axis could be determined as a potential mediator in liver macrophages and cirrhosis progression.

## 2. Materials and methods

### 2.1. Data resources and DEG analysis

In this study, expression profile datasets from bulk RNA-sequencing and single-cell RNA (scRNA)-sequencing analyses were downloaded from the Gene Expression Omnibus (GEO) database (http://www.ncbi.nlm.nih.gov/geo). Basic information is listed in Table 1. Probes from the raw data were matched with the official gene symbols with the DAVID online tool (https://david.ncifcrf.gov/)[8] and noncoding RNAs were excluded. Differentially expressed gene (DEG)-related analyses were performed using R Studio (limma package) [9]. Fold change (FC) >2 and *p* < 0.01 were used as the statistical criteria to identify significant DEGs. In addition, overlapping gene clusters were identified by the generation of Venn diagrams (http://bioinformatics.psb.ugent.be/webtools/Venn/) [10].

**Table 1.** Human dataset resources

GEO series	Experiment	Platform	Overall design
GSE164760	Bulk RNA-seq	GPL13667	74 NASH livers, 8 cirrhotic livers, and 6 healthy livers
GSE89632	Bulk RNA-seq	GPL14951	19 NASH livers, 20 steatotic livers, and 24 healthy livers
GSE49541	Bulk RNA-seq	GPL570	40 mild fibrotic NASH liver and 32 advaced fibrotic NASH
GSE139602	Bulk RNA-seq	GPL13667	5 fibrosis (eCLD), 8 compensated cirrhosis, 12 decompensated cirrhosis, 8 ACLF, and 6 control healthy livers
GSE123661	scRNA-seq	GPL17303	4 cirrhotic and 5 healthy KC samples
GSE136103	scRNA-seq	GPL20301	5 healthy livers, 5 cirrhotic livers, and 4 PBMC samples
GSE98782	scRNA-seq	GPL17021	2 MoMFs and 2 KCs from chronic injured livers

### 2.2. Immune cell infiltration analysis and dataset regrouping

To investigate immune cell infiltration, raw data from bulk RNA-seq datasets were included and evaluated by multiple computational methods. The xCell package (https://xcell.ucsf.edu/) [11] in R Studio was applied, and the results were compared and validated with the CIBERSORT (https://cibersort.stanford.edu) [12] and EPIC (https://github.com/GfellerLab/EPIC) [13] methods. Then, raw data from 74 NASH liver samples in the GSE164760 dataset were classified into the high MP and low MP groups based on the cell-type enrichment scores from Xcell. Thereby, DEG-related analyses were performed using R Studio (limma package).

### 2.3. Weighted gene co-expression network analysis (WGCNA)

To outline the gene expression patterns in multiple samples, the WGCNA package (http://www.genetics.ucla.edu/labs/horvath/CoexpressionNetwork/Rpackages/WGCNA), which is a comprehensive method used to cluster co-expressed genes and compare the associations between modules and specific traits, was used with R Studio [14]. The expression matrix of NASH samples in the GSE164760 dataset regrouped by MP level was analyzed by WGCNA. The correlation analysis implemented in the WGCNA package is based on the Pearson method, and specific parameters (min Module Size = 30, reassign Threshold = 0, and merge Cut Height = 0.25) were used to run the program. The clusters of co-expressed genes were then displayed as modules labeled in multiple colors, with correlation coefficient values and significance (*p* values).

### 2.4. Functional enrichment and protein–protein interaction (PPI) analysis

Functional enrichment was analyzed and classified primarily according to Kyoto Encyclopedia Genes and Genomes (KEGG) pathways [15] and Gene Ontology (GO) terms [16]. With the KEGG Orthology-Based Annotation System (KOBAS) 3.0 web server (http://kobas.cbi.pku.edu.cn/kobas3) [17] and Gene Set Enrichment Analysis (GSEA) software (https://www.gsea-msigdb.org/gsea) [18], highly relevant functional clusters were identified, and data were plotted. PPI analysis was conducted with the STRING web tool (https://string-db.org/) [19], and the topological network was then constructed with Cytoscape software (version 3.8.2) [20]. Then, hub clusters and genes were identified using the MCODE plugin (degree cutoff = 2, node score cutoff = 0.2, and K-cor = 2).

### 2.5. Identification of miRNA–protein interactions

Databases (MirWalk: www.mirwalk.umm.uni-heidelberg.de^21^; TargetScan: www.targetscan.org^22^; mirDB: www.mirdb.org [23]; and miRTarBase: www.mirtarbase.cuhk.edu.cn) [24] were used for prediction of miRNA–mRNA interactions. Associated miRNA alternatives were screened out by inputting mRNA targets. Results were filtered with *p* < 0.05 and downloaded from websites.

### 2.6. Cell culture and treatment

The human monocyte line THP-1 was purchased from the National Collection of Authenticated Cell Cultures (Shanghai, China) and cultured in RPMI 1640 medium supplied with 10% FBS (Gibco, Australia) in an incubator with 5% CO_2_ at 37°C. Macrophage differentiation of THP-1 monocytes was induced with 100 nmol/L phorbol 12-myristate-13-acetate (PMA) (Sigma–Aldrich, USA) for 48 h. Palmitic acid (PA) and oleic acid (OA) (both from Sigma–Aldrich, USA) were dissolved at a 2:1 ratio (final concentration of 30 µM) in RPMI 1640 medium supplemented with 0.1 M NaOH and 0.1% BSA (both from Sigma–Aldrich, USA) [25].

### 2.7. RNA quantification

Total RNA was extracted from cultured cells using TRIzol® reagent (Invitrogen; Thermo Fisher Scientific, USA). Reverse transcription was conducted with a PrimeScript™ RT Reagent Kit (Takara Biotechnology Co., Ltd.), and real-time quantification was subsequently performed using a SYBR® Premix Ex Taq Kit (Takara Biotechnology Co., Ltd.). The relative RNA expression levels were calculated using the 2^‑ΔΔCt^ method [26]. The primer pairs used in this study are listed in Supplementary Table 1.

### 2.8. Statistical analysis

SPSS 26.0 (SPSS Statistics, USA) was used for general statistical analysis. GraphPad Prism 9.0 software (GraphPad Software, USA) and R Studio were used to generate plots. Significant differences were identified using one‑way ANOVA with the Bonferroni post hoc test. Pearson correlation analysis was performed to calculate correlation coefficients. Data are presented as the mean ± SEM values. *p* < 0.05 indicated a statistically significant difference (labeled ‘*’).

## 3. Results

We hypothesized that miR-4715-3p/*GSDMD* axis may play as a mediator for macrophage infiltration and NAFLD progression. Multiple RNA-seq datasets were included in this study, and therefore, immune infiltration and hub genes were determined. *GSDMD* was identified as the key factor both associated with high macrophage infiltration and NAFLD progression. Predicted as a regulator to *GSDMD*, miR-4715-3p was validated in FFA-induced macrophage injury, which was significantly correlated with *GSDMD*.

### 3.1. Identification of DEGs and immune cell landscape in NASH livers

**Figure 1.**
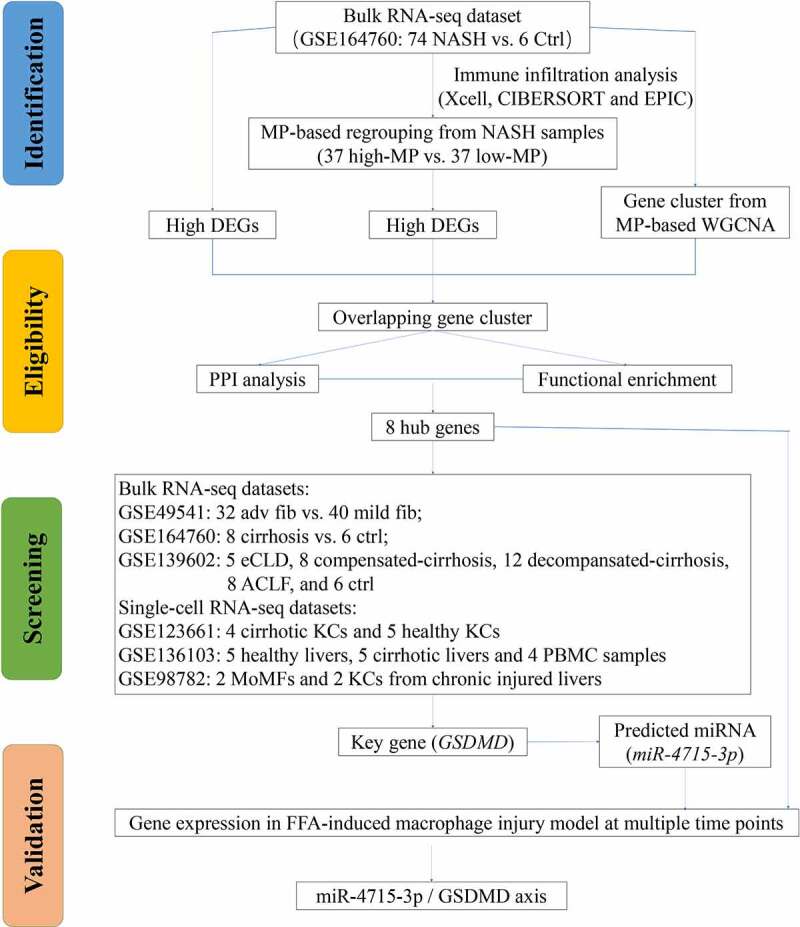
PRISMA flow.

**Figure 2.**
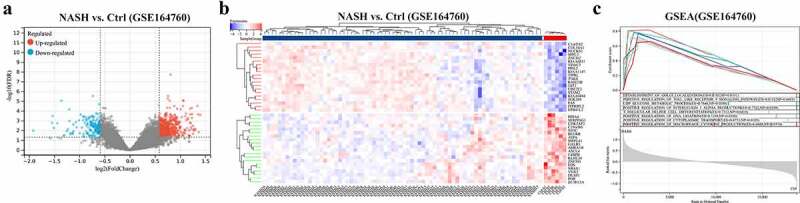
(a) Both significantly up- and downregulated DEGs were screened out from GSE164760 and displayed in the volcano plot. (b) Top significantly up- and downregulated genes were displayed in a heatmap. (c) Functional enrichment was analyzed with GSEA. *p* < 0.05 is regarded as statistical significance.

The overall flow chart of the technical paths followed in this study is shown in Figure 1. In 74 NASH and 6 healthy control liver samples, DEGs were identified as genes with |log2 (fold change)| > 1.5 and *p* < 0.05 (Figure 2a); the DEGs comprised 444 upregulated genes and 83 downregulated genes. The top-ranked DEGs were displayed in a clustering heatmap. The top 10 upregulated genes were *C1orf162, COL10A1, NUCKS1, SPPL3, ZNF362, KIAA0831 (ATG14), VDAC3, PPIL2, KIAA1147* (*DENND11*), and *TIPLR*. The top 10 downregulated genes were *PDIA4, SERPING1, CDK2AP2, C19orf60, NT5C, BLVRB, ASPA, MRPL41, GALR3,* and *ADRA1B*. GSEA was then used to perform functional enrichment analysis on the upregulated gene cluster and visualize the results. Among the leading enrichment patterns, biological processes associated with immune cells (Th cells and macrophages) were revealed to be statistically significant (Figure 2c).

**Figure 3.**
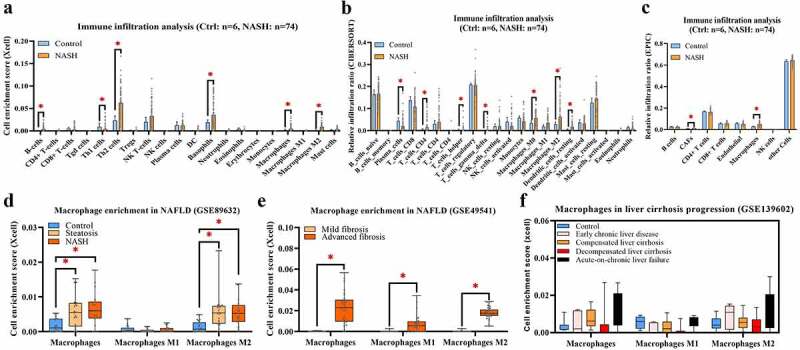
Immune infiltration of GSE164760 is assessed with Xcell (a), CIBERSORT (b), and EPIC (c). Enrichment of MP clusters (total MP, M1-MP, and M2-MP) from GSE89632 (d), GSE49541 (e), and GSE139602 (f) are shown in plots. *p* < 0.05 (‘*’) is regarded as statistical significance.

Accordingly, Xcell, CIBERSORT, and EPIC were applied to investigate and compare immune cell infiltration in NASH. Excluding nonimmune cells, the main myeloid cells and lymphocytes were evaluated and displayed. As shown in Figure 3a, the infiltration levels of B cells, Th2 cells, basophils, MPs, and M2-MPs were significantly increased in the NASH group compared with the control group. In contrast, the infiltration level of Th1 cells was decreased. As shown in Figure 3b, the infiltration levels of CD4+ T cells, T helper cells, gamma delta T cells, M0-MPs, and M2-MPs were significantly increased in the NASH group compared with the control group. In contrast, the infiltration level of plasma cells was decreased. As shown in Figure 3c, the infiltration levels of fibroblasts and MPs were increased. These findings conclusively show that MP infiltration is commonly elevated in NASH livers compared with healthy livers. To further explore the correlations between MP infiltration and cirrhosis progression in NAFLD, MP clusters (total MPs, M1-MPs, and M2-MPs) were evaluated with xCell in multiple RNA-seq datasets (GSE89632, GSE49541, and GSE139602). Increasing trends in infiltration were found for both steatosis (Figure 3d) and cirrhosis progression (Figure 3e and f).

### 3.2. The identification of macrophage - associated hub genes in NAFLD progression

**Figure 4.**
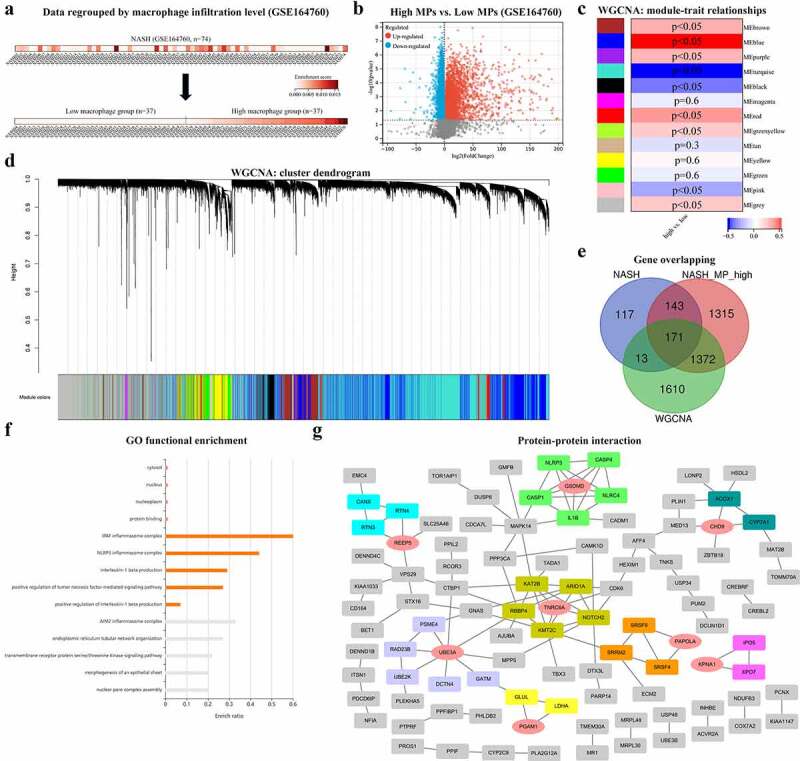
(a) NASH samples of GSE164760 are grouped with MP ES from Xcell (low MP: ES = 0 and high MP >0). (b) Both significantly up- and downregulated DEGs were screened out from regrouped NASH samples of GSE164760 and displayed in the volcano plot. MP-associated gene cluster is selected with WGCNA. Module–trait relationships (c) and cluster dendrogram (d) are shown in charts. (e) 171 overlapping genes are generated from high DEGs of GSE164760 and regrouped NASH samples, as well as WGCNA gene cluster. GO enrichment analysis (f) and PPI analysis (g) are carried out from 171 overlapping genes. Hub genes are labeled in red and round shape. *p* < 0.05 is regarded as statistical significance.

Based on the xCell scores of MPs, 74 NASH liver samples from GSE164760 were divided into the high-MP (*n* = 37) and low-MP (*n* = 37) groups (Figure 4a). The DEGs were then sorted and are shown in Figure 4b. In addition, WGCNA was conducted to generate gene coexpression modules, from which the module–trait relationships (Figure 4c) and cluster dendrograms (Figure 4d) were generated. Considering both the correlation coefficients and *p* values, the MEblue module, with 3166 genes, was identified. After overlapping three gene clusters, 171 genes were identified as the most significantly upregulated macrophage-associated genes in NASH livers (Figure 4e). Then, GO enrichment analysis was carried out with these 171 genes, and the results are shown in Figure 4f; the terms NLRP3 inflammasome, interleukin-1 beta (*IL-1β*), and tumor necrosis factor (*TNF*) were found to be significantly enriched. A PPI network was constructed to visualize functional interactions. Hub clusters and seed genes were also identified (Figure 4g).

**Figure 5.**
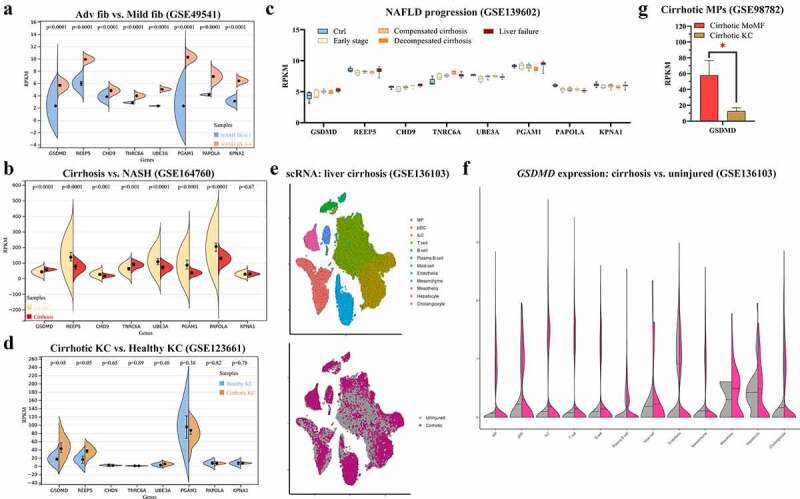
Expressions of eight hub genes are compared, respectively, from GSE49541 (a), GSE1647560 (b), GSE139602 (c), and GSE123661 (d). (e) Distribution of cell populations and disease conditions are generated from GSE136103 with tSNE. (f) Expression of *GSDMD* is shown in multiple cell populations from GSE136103. *p* < 0.05 is regarded as statistical significance.

To assess clinical correlations, the expression levels of eight hub/seed genes (*GSDMD, REEP5, CHD9, TNRC6A, UBE3A, PGAM1, PAPOLA*, and *KPNA1*) were verified on bulk RNA-seq datasets (GSE49541, GSE164760, and GSE139602) and single-cell RNA-seq datasets (GSE123661, GSE136103, and GSE98782). All eight genes were upregulated in NASH livers at advanced fibrosis stages compared with mild fibrosis stages (Figure 5a). In contrast to NASH livers, *GSDMD* and *TNRC6A* were upregulated in cirrhotic livers (Figure 5b). Among all stages of progression, a continuously increasing trend in *GSDMD* and *TNRC6A* expression was identified (Figure). At the single-liver MP level, *GSDMD* was significantly upregulated in cirrhotic KCs (Figure 5d). The distribution of cell populations in NAFLD-related cirrhosis is shown in Figure 5e. After single-cell analysis, *GSDMD* was found to be upregulated in the liver MP population (Figure 5f). Moreover, among macrophages in cirrhotic livers, monocyte-derived macrophages (MoMFs) expressed more *GSDMD* than KCs (Figure 5g). These results indicate that *GSDMD* may not only play crucial roles as a biomarker for NAFLD progression but also function through liver macrophages.

### 3.3. *Correlative upregulation of* miR-4715-3p *and* GSDMD *in FFA-induced macrophage injury*

**Figure 6.**
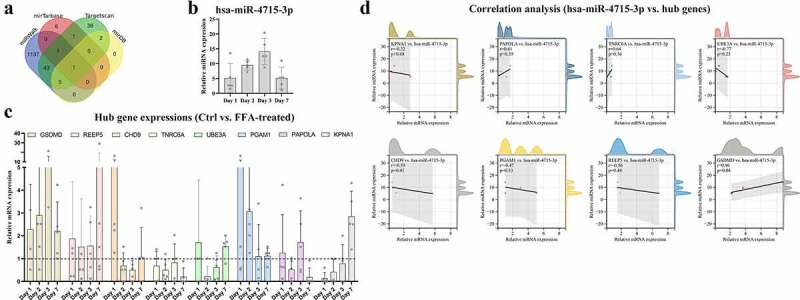
(a) *hsa-miR-4715-3p* is screened out from *GSDMD*-associated miRNAs. Expressions of *hsa-miR-4715-3p* (b) and eight hub genes (c) in FFA-treated THP1-derived macrophages are displayed. (d) Pearsoncorrelation analysis is conducted,respectively, between *hsa-miR-4715-3p* and eight hub genes. *p* < 0.05 is regarded as statistical significance.

By searching an online database, multiple miRNA clusters highly correlated with *GSDMD* were identified. Then, hsa-miR-4715-3p was found in all miRNA clusters (Figure 6a). To mimic immune modulation in NAFLD, an in vitro model of FFA-induced macrophage injury was applied. Then, THP-1 cells were treated sequentially with PMA to induce differentiation into macrophages and with FFA solution for 1 week (the cytotoxicity of FFA in THP-1-derived macrophages is shown in Supplementary Figure 1). MoMFs were harvested at sequential time points (on the first, second, third, and seventh days) for RNA quantification. The expression levels of hsa-miR-4715-3p (Figure 6b) and the eight hub genes (Figure 6c) are shown at different time points. hsa-miR-4715-3p and *GSDMD* were generally upregulated after FAA treatment, and their expression peaked on the third day. In contrast, only *REEP5* was upregulated at all time points. Therefore, Pearson correlation analysis was conducted on hsa-miR-4715-3p and the hub genes (Figure 6d), and *GSDMD* expression was found to be significantly positively correlated with hsa-miR-4715-3p expression (*R* = 0.96, *p* = 0.04). The results indicate that co-expression of miR-4715-3p and *GSDMD* is significantly induced in response to FFA stimulation in macrophages.

## 4. Discussion

NASH is considered an inflammatory subtype of NAFLD with steatosis and evidence of hepatocyte injury and multiple immune cell interactions [27]. Furthermore, fibrosis consistently occurs with hepatic cell injury and gradually progresses to cirrhosis, which results in hepatocarcinoma and liver failure [28]. Generally, steatosis and liver function can be measured by serological and imaging approaches. However, liver fibrosis could historically be assessed only by biopsy, which is sensitive and accurate but results in unavoidable injury and pain in patients [29]. Currently, the most effective noninvasive methods are ultrasonic transient elastography (for example, fiber scanning) and magnetic resonance elastography. Moreover, noninvasive estimation systems can evaluate the fibrosis stage in patients without biopsy, with the NAFLD fibrosis score and fibrosis-4 index (FIB-4) commonly used. The complex pathophysiological mechanism of NASH progression remains unrevealed [30,31]. Currently, multiple therapies aimed at several targets, such as changes in the microbiome and intestinal permeability, oxidative stress, insulin resistance, apoptosis, lipotoxicity, inflammation, bile acid metabolism, and fibrogenesis. Given that the therapeutic effects of traditional drugs on NASH progression are poor, the discovery of disease targets and research on targeted therapies are of great importance [32]. Generally, liver macrophages (including KCs and MoMFs) play pivotal roles in the progression and resolution of fibrosis. With the development of fibrosis, injury-induced inflammation further enhances macrophage recruitment and HSC activation [33]. In addition, a variety of matrix metalloproteinases (MMP-9, MMP-12, and MMP-13) secreted by liver macrophages participate in matrix degradation, thus contributing to the alleviation of liver injury and fibrosis [34,35].

The initial goal of this study was to characterize immune cell infiltration and identify progression-related mediators in NAFLD. Bulk and single-cell RNA sequencing datasets of NASH and fibrosis progression were introduced as basic data sources. Then, multiple bioinformatic methods and databases were used to reveal the immune cell landscape and important factors. Of note, a novel method of dataset regrouping was developed in this study based on xCell and WGCNA to identify macrophage-associated DEGs. Subsequently, hub genes were identified from the PPI network. Further screening and validation were carried out with single-cell RNA-seq datasets and a model of FFA-induced macrophage injury. As a result, GSDMD and hsa-miR-4715-3p were found to be co-upregulated, indicating NAFLD progression.

GSDMD is a key pyroptotic substrate of inflammation-triggered caspase modules. GSDMD initiates pyroptosis directly via caspase-1-induced cleavage. Moreover, GSDMD-related pyroptosis mainly occurs in macrophages and is directly mediated by inflammasomes [36,37]. In addition to pyroptotic factors (caspase-1/-4 and IL-1β), NLRP3 and NLRC4, which act as classical regulators of inflammasomes [38], were also included in the final gene cluster. Accordingly, inflammasome-induced pyroptosis is considered a crucial macrophage-fate-accompanying NAFLD progression. In addition, miRNA–protein interaction analysis revealed that hsa-miR-4715-3p might act as a potential upstream regulator of GSDMD. Previous studies have revealed multiple roles and function of several microRNAs in NAFLD and liver fibrosis [39–42]. According to a literature review, hsa-miR-4715-3p has been reported in only a few cancer studies [43,44]. In conclusion, the miR-4715-3p/GSDMD axis may be a novel signaling pathway that mediates not only macrophage functions but also NAFLD progression. Although this study provided robust evidence at the transcriptome level, further investigations are required to validate the functions of the GSDMD protein as an executor of inflammasome-mediated pyroptosis.

However, this study is mostly based on RNA-seq datasets and computational analysis, leading to inevitable limitations. Baseline levels and batch effects of different clinical cohorts could not be entirely adjusted, referring to statistical biases. Moreover, molecular validations were included but not emphasized in this study. Hence, to determine the exact relationship between GSDMD and hsa-miR-4715-3p, more molecular studies are required.

## 5. Conclusions

During fibrosis progression in NAFLD, the macrophage infiltration and highly associated gene – GSDMD – are elevated. The hsa-miR-4715-3p is upregulated in FFA-induced macrophage injury and parallelly altered by GSDMD expression. Thus, the macrophage-associated miR-4715-3p/GSDMD axis potentially indicates fibrosis progression in NAFLD.

## Supplementary Material

Supplemental Material

## Data Availability

The datasets used and analyzed in the current study are available from the corresponding author on reasonable request.

## References

[1] Byrne CD, Targher G. NAFLD: a multisystem disease. J Hepatol. 2015;62:S47–64. 25920090 10.1016/j.jhep.2014.12.012

[2] Cobbina E, Akhlaghi F. Non-alcoholic fatty liver disease (NAFLD) – pathogenesis, classification, and effect on drug metabolizing enzymes and transporters. Drug Metab Rev. 2017;49:197–211. 28303724 10.1080/03602532.2017.1293683 PMC5576152

[3] Eslam M, Valenti L, Romeo S. Genetics and epigenetics of NAFLD and NASH: clinical impact. J Hepatol. 2018;68:268–279. 29122391 10.1016/j.jhep.2017.09.003

[4] Tacke F, Zimmermann HW. Macrophage heterogeneity in liver injury and fibrosis. J Hepatol. 2014;60:1090–1096. 24412603 10.1016/j.jhep.2013.12.025

[5] Wynn TA, Vannella KM. Macrophages in tissue repair, regeneration, and fibrosis. Immunity. 2016;44:450–462. 26982353 10.1016/j.immuni.2016.02.015 PMC4794754

[6] Udhaya Kumar S, Thirumal Kumar D, Bithia R, et al. Analysis of differentially expressed genes and molecular pathways in familial hypercholesterolemia involved in atherosclerosis: a systematic and bioinformatics approach. Front Genet. 2020;11:734. 32760426 10.3389/fgene.2020.00734 PMC7373787

[7] Udhaya Kumar S, Saleem A, Thirumal Kumar D, et al. A systemic approach to explore the mechanisms of drug resistance and altered signaling cascades in extensively drug-resistant tuberculosis. Adv Protein Chem Struct Biol. 2021;127:343–364. 34340773 10.1016/bs.apcsb.2021.02.002

[8] Huang da W, Sherman BT, Lempicki RA. Systematic and integrative analysis of large gene lists using DAVID bioinformatics resources. Nat Protoc. 2009;4:44–57. 19131956 10.1038/nprot.2008.211

[9] Ritchie ME, Phipson B, Wu D, et al. limma powers differential expression analyses for RNA-sequencing and microarray studies. Nucleic Acids Res. 2015;43:e47. 25605792 10.1093/nar/gkv007 PMC4402510

[10] Jia A, Xu L, Wang Y. Venn diagrams in bioinformatics. Brief Bioinform. 2021;22(5):bbab108. ISBN: 1477-4054. 33839742 10.1093/bib/bbab108

[11] Aran D, Hu Z, Butte AJ. xCell: digitally portraying the tissue cellular heterogeneity landscape. Genome Biol. 2017;18:220. 29141660 10.1186/s13059-017-1349-1 PMC5688663

[12] Chen B, Khodadoust MS, Liu CL, et al. Profiling tumor infiltrating immune cells with CIBERSORT. Methods Mol Biol. 2018;1711:243–259. 29344893 10.1007/978-1-4939-7493-1_12 PMC5895181

[13] Racle J, Gfeller D. EPIC: a tool to estimate the proportions of different cell types from bulk gene expression data. Methods Mol Biol. 2020;2120:233–248. 32124324 10.1007/978-1-0716-0327-7_17

[14] Langfelder P, Horvath S. WGCNA: an R package for weighted correlation network analysis. BMC Bioinformatics. 2008;9:559. 19114008 10.1186/1471-2105-9-559 PMC2631488

[15] Kanehisa M, Furumichi M, Tanabe M, et al. KEGG: new perspectives on genomes, pathways, diseases and drugs. Nucleic Acids Res. 2017;45:D353–d61. 27899662 10.1093/nar/gkw1092 PMC5210567

[16] Carbon, S , Thomas, P.D , Albou, L.P *et al*. The gene ontology resource: 20 years and still GOing strong. Nucleic Acids Res. 2019;47:D330–d8. 30395331 10.1093/nar/gky1055 PMC6323945

[17] Wu J, Mao X, Cai T, et al. KOBAS server: a web-based platform for automated annotation and pathway identification. Nucleic Acids Res. 2006;34:W720–4. 16845106 10.1093/nar/gkl167 PMC1538915

[18] Subramanian A, Tamayo P, Mootha VK, et al. Gene set enrichment analysis: a knowledge-based approach for interpreting genome-wide expression profiles. Proc Natl Acad Sci U S A. 2005;102:15545–15550. 16199517 10.1073/pnas.0506580102 PMC1239896

[19] Szklarczyk D, Gable AL, Nastou KC, et al. The STRING database in 2021: customizable protein-protein networks, and functional characterization of user-uploaded gene/measurement sets. Nucleic Acids Res. 2021;49:D605–d12. 33237311 10.1093/nar/gkaa1074 PMC7779004

[20] Shannon P, Markiel A, Ozier O, et al. Cytoscape: a software environment for integrated models of biomolecular interaction networks. Genome Res. 2003;13:2498–2504. 14597658 10.1101/gr.1239303 PMC403769

[21] Dweep H, Gretz N, Sticht C. miRWalk database for miRNA-target interactions. Methods Mol Biol. 2014;1182:289–305. 25055920 10.1007/978-1-4939-1062-5_25

[22] Riffo-Campos ÁL, Riquelme I, Brebi-Mieville P. Tools for sequence-based miRNA target prediction: what to choose? Int J Mol Sci. 2016;17:1987. 10.3390/ijms17121987 PMC5187787 27941681

[23] Chen Y, Wang X. Wang X. miRDB: an online database for prediction of functional microRNA targets. Nucleic Acids Res. 2020;48:D127–d31. 31504780 10.1093/nar/gkz757 PMC6943051

[24] Huang HY, Lin YC, Li J, et al. miRTarBase 2020: updates to the experimentally validated microRNA-target interaction database. Nucleic Acids Res. 2020;48:D148–d54. 31647101 10.1093/nar/gkz896 PMC7145596

[25] Liu H, Kai L, Du H, et al. LPS inhibits fatty acid absorption in enterocytes through TNF-α secreted by macrophages. Cells. 2019;8(12):1626. 10.3390/cells8121626 PMC6953048 31842409

[26] Hawkins SFC, Guest PC. Multiplex analyses using real-time quantitative PCR. Methods Mol Biol. 2017;1546:125–133. 27896761 10.1007/978-1-4939-6730-8_8

[27] Younossi Z, Anstee QM, Marietti M, et al. Global burden of NAFLD and NASH: trends, predictions, risk factors and prevention. Nat Rev Gastroenterol Hepatol. 2018;15:11–20. 28930295 10.1038/nrgastro.2017.109

[28] Chalasani N, Younossi Z, Lavine JE, et al. The diagnosis and management of nonalcoholic fatty liver disease practice guidance from the American Association for the Study of Liver Diseases. Hepatology. 2018;67:328–357. 28714183 10.1002/hep.29367

[29] Pai RK, Kleiner DE, Hart J, et al. Standardising the interpretation of liver biopsies in non-alcoholic fatty liver disease clinical trials. Aliment Pharmacol Ther. 2019;50:1100–1111. 31583739 10.1111/apt.15503 PMC6817398

[30] Sheka AC, Adeyi O, Thompson J, et al. Nonalcoholic steatohepatitis: a review. Jama. 2020;323:1175–1183. 32207804 10.1001/jama.2020.2298

[31] Di Mauro S, Scamporrino A, Filippello A, et al. Clinical and molecular biomarkers for diagnosis and staging of NAFLD. Int J Mol Sci. 2021;23:22. 34769333 10.3390/ijms222111905 PMC8585051

[32] Parola M, Pinzani M. Liver fibrosis: pathophysiology, pathogenetic targets and clinical issues. Mol Aspects Med. 2019;65:37–55. 30213667 10.1016/j.mam.2018.09.002

[33] Iredale JP. Models of liver fibrosis: exploring the dynamic nature of inflammation and repair in a solid organ. J Clin Invest. 2007;117:539–548. 17332881 10.1172/JCI30542 PMC1804370

[34] Dou L, Shi X, He X, et al. Macrophage phenotype and function in liver disorder. Front Immunol. 2019;10:3112. 32047496 10.3389/fimmu.2019.03112 PMC6997484

[35] Kazankov K, Jørgensen SMD, Thomsen KL, et al. The role of macrophages in nonalcoholic fatty liver disease and nonalcoholic steatohepatitis. Nat Rev Gastroenterol Hepatol. 2019;16:145–159. 30482910 10.1038/s41575-018-0082-x

[36] Kayagaki N, Stowe IB, Lee BL, et al. Caspase-11 cleaves gasdermin D for non-canonical inflammasome signalling. Nature. 2015;526:666–671. 26375259 10.1038/nature15541

[37] Shi J, Zhao Y, Wang K, et al. Cleavage of GSDMD by inflammatory caspases determines pyroptotic cell death. Nature. 2015;526:660–665. 26375003 10.1038/nature15514

[38] Rathinam VA, Fitzgerald KA. Inflammasome complexes: emerging mechanisms and effector functions. Cell. 2016;165:792–800. 27153493 10.1016/j.cell.2016.03.046 PMC5503689

[39] Xu A, Zhong G, Wang J, et al. MicroRNA 200a inhibits liver fibrosis of schistosoma. Bioengineered. 2021;12:4736–4746. 34338152 10.1080/21655979.2021.1950441 PMC8806541

[40] Calvente CJ, Tameda M, Johnson CD, et al. Neutrophils contribute to spontaneous resolution of liver inflammation and fibrosis via microRNA-223. J Clin Invest. 2019;129:4091–4109. 31295147 10.1172/JCI122258 PMC6763256

[41] Zhang T, Hu J, Wang X, et al. MicroRNA-378 promotes hepatic inflammation and fibrosis via modulation of the NF-κB-TNFα pathway. J Hepatol. 2019;70:87–96. 30218679 10.1016/j.jhep.2018.08.026 PMC6554744

[42] Yu Y, Tian T, Tan S, et al. MicroRNA-665-3p exacerbates nonalcoholic fatty liver disease in mice. Bioengineered. 2022;13:2927–2942. 35038955 10.1080/21655979.2021.2017698 PMC8973643

[43] Gomaa A, Peng D, Chen Z, et al. Epigenetic regulation of AURKA by miR-4715-3p in upper gastrointestinal cancers. Sci Rep. 2019;9:16970. 31740746 10.1038/s41598-019-53174-6 PMC6861278

[44] Yu W, Yao J, Lyu P, et al. XPG is modulated by miR-4715-3p and rs873601 genotypes in lung cancer. Cancer Manag Res. 2021;13:3417–3427. 33907465 10.2147/CMAR.S294365 PMC8064622

