# Supplementary material for: The macrophage-associated microRNA-4715-3p / Gasdermin D axis potentially indicates fibrosis progression in nonalcoholic fatty liver disease: evidence from transcriptome and biological data
Source: Bioengineered. 2022 May 6;13(5):11740–51. doi: 10.1080/21655979.2022.2072602 (PMC9275955; doi:10.1080/21655979.2022.2072602)
Supplement: Supplemental Material [file KBIE_A_2072602_SM3753.zip › supplementary/Supplementary Table 1.docx]

| **Table1: human dateset resources** | | |  |
| --- | --- | --- | --- |
| **GEO series** | **Experiment** | **Platform** | **Overall design** |
| GSE164760 | Bulk RNA-seq | GPL13667 | 74 NASH livers, 8 cirrhotic livers and 6 healthy livers |
| GSE89632 | Bulk RNA-seq | GPL14951 | 19 NASH livers, 20 steatotic livers and 24 healthy livers |
| GSE49541 | Bulk RNA-seq | GPL570 | 40 mild fibrotic NASH liver and 32 advaced fibrotic NASH |
| GSE139602 | Bulk RNA-seq | GPL13667 | 5 fibrosis(eCLD), 8 compensated cirrhosis, 12 decompensated cirrhosis, 8 ACLF, and 6 control healthy livers |
| GSE123661 | scRNA-seq | GPL17303 | 4 cirrhotic and 5 healthy KC samples |
| GSE136103 | scRNA-seq | GPL20301 | 5 healthy livers, 5 cirrhotic livers and 4 PBMC samples |
| GSE98782 | scRNA-seq | GPL17021 | 2 MoMFs and 2 KCs from chronic injured livers |
