# Supplementary figures and images for: The macrophage-associated microRNA-4715-3p / Gasdermin D axis potentially indicates fibrosis progression in nonalcoholic fatty liver disease: evidence from transcriptome and biological data
Source: Bioengineered. 2022 May 6;13(5):11740–51. doi: 10.1080/21655979.2022.2072602 (PMC9275955; doi:10.1080/21655979.2022.2072602)

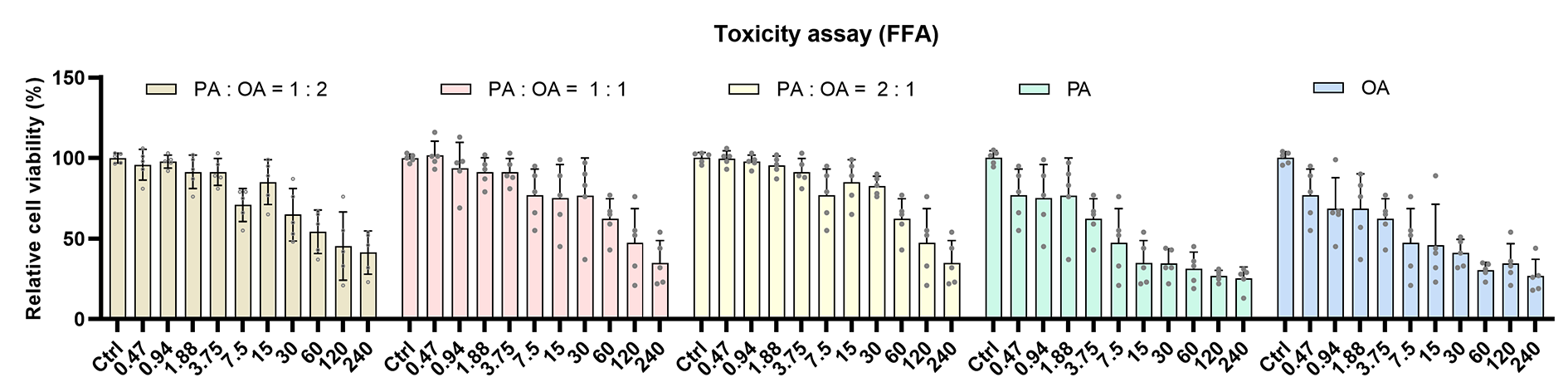

Supplement: Supplemental Material [file KBIE_A_2072602_SM3753.zip › supplementary/supplementary Figure1.tif]
